# The Search for and Presence of Calling: Latent Profiles and Relationships With Work Meaning and Job Satisfaction

**DOI:** 10.3389/fpsyg.2021.633351

**Published:** 2021-02-23

**Authors:** Feifei Li, Runkai Jiao, Dan Liu, Hang Yin

**Affiliations:** ^1^School of Psychology, Northeast Normal University, Changchun, China; ^2^National Training Center for Kindergarten Principals, Ministry of Education, Northeast Normal University, Changchun, China

**Keywords:** search for calling, presence of calling, work meaning, job satisfaction, latent profile analysis

## Abstract

Previous studies showed inconsistent results on the association between searching for calling and its psychosocial functioning outcomes (i.e., work meaning and job satisfaction). The link of searching for calling to its psychosocial functioning outcomes may be influenced by the presence of calling because the search for and presence of calling can co-exist within individuals. Thus, the present study employed a person-centered method (latent profile analysis) to identify subgroups combining the search for and presence of a calling and then explore the identified profiles' differences in work meaning and job satisfaction. Study participants were Chinese kindergarten teachers (*n* = 726). Latent profile analysis revealed four different groups: (1) actively maintaining calling (24.93%), (2) unsustainable calling (11.43%), (3) moderately increasing calling (23.14%), and (4) actively increasing calling (40.50%). Subsequent analyses showed notable differences across the four groups on work meaning and job satisfaction. Participants in profile 1 with both the highest searching for and presence of calling would experience more work meaning and job satisfaction than those in the other profiles whose strengths of searching for and presence of calling were relatively low. Participants in profile 4 had higher searching for and presence of calling than those in profile 3, and they experienced more meaningfulness at work and were more satisfied with their job. These findings indicate that actively searching for calling is closely associated with more work meaning and job satisfaction among people who already perceive intensive calling. Implications, limitations, and future directions of the results are discussed.

## Introduction

Human motivation is an important area to interpret individual differences, on which different scholars proposed different theoretical positions. For instance, Freud put forward a seminal theoretical position from a Psychoanalytical perspective, considering the unconscious desires and seeking gratification of innate instinctual drives as the primary motivation of all human functioning (Willmott et al., 2018). Alternatively, Roger took more consideration for humans' roles as active agents within their own experiences and motivations throughout the life-course than Freud's positions. He suggested that all people are primarily motivated by a need to fulfill their potential and achieve self-actualization (Willmott et al., 2018). Roger's theoretical position has been recognized by many scholars and has gained massive development over the years. Thereinto, Self-Determination Theory (SDT), proposed by Deci and Ryan (2000), is the most representative and influential, emphasizing that people have inherent tendencies toward psychological growth, integrity, and self-actualization (Koole et al., 2019).

Given that work has been considered as a vital context for actualizing personal potential or growth (Brown and Lent, 2016), the idea of work as a calling has received considerable attention in the career development and organization domain. A sense of calling reflects one's psychological approach to work, which is characterized by a transcendent summons or guide force, the alignment of their career with a broader sense of meaning and purpose in life, and an intention to help others or advance the greater good via their career (Duffy and Dik, 2013; Zhang et al., 2018; Dik and Shimizu, 2019). One person could be searching for a calling or perceiving a calling. Perceiving a calling refers to the idea that someone has identified a calling-oriented work and perceives themselves as currently having a calling; Searching for a calling refers to the idea that someone is intensively and actively seeking to find or augment their experiences of calling (Dik et al., 2012; Autin et al., 2017). Substantial studies focus on the presence of calling and find its close association with numerous positive outcomes (e.g., career maturity, work engagement, and well-being; for a review, see Duffy and Dik, 2013). Moreover, the presence of calling is closely related to a fully functioning person's characteristics. Prior studies considered the presence of a calling as the convergence of individuals' sense of what they would like to do, should do, and actually do, suggesting that people perceiving intensive calling would feel that they are developing their full potential and expressing the real self-through work (Elangovan et al., 2010). Compared with research on the presence of calling, searching for a calling is mostly related to adverse outcomes (Dobrow et al., 2019) and draws relatively little attention. However, the search for calling is essential for the presence of calling. No one was born with a clear perception of their calling, and they need to search for it. Further, the development of calling is an ongoing process rather than a one-time event. Even though people find or discern their calling, they need continually evaluate their career and seek ways to maintain or enhance the presence of calling instead of ceasing the search (Dik and Duffy, 2009; Dik et al., 2012). Some researchers even suggested searching for and presence of calling as two overarching and overlapping aspects of the calling construct (Dik et al., 2012). Thus, searching for calling needs further empirical research as it is an essential stage in developing one's calling and represents a motivation toward the pursuit of self-actualization.

## The Outcomes of Searching for Calling

The existing studies focused relatively more on exploring the outcomes of the search for calling. However, conflicting results exist regarding whether the search for calling positively or negatively associates with people's psychosocial functioning outcomes. Searching for meaning (SOM) and presence of meaning (POM) have been seen as the essential components of searching for and presence of calling, respectively (Dik and Duffy, 2009; Dik et al., 2012). Analogous to SOM and POM (Steger et al., 2008), the search for calling reflects the motivational dimension of calling, and the presence of calling reflects the cognitive dimension of calling. Because having a calling is a key element of self-actualized people (Thompson and Bunderson, 2019), the search for calling represents a vital function-like human motivation, which motivates people toward achieving self-actualization. From this perspective, actively searching for calling might positively associate with people's healthy functioning. On the contrary, some scholars considered the search for calling as a process of resource depletion, which yields adverse outcomes (e.g., depression, indecisiveness; Duffy and Sedlacek, 2007; Steger and Dik, 2009; Dobrow et al., 2019). Prior empirical studies also showed some contradictory results. For instance, work meaning and job satisfaction have been posited as typical consequences of developing a calling (e.g., Duffy et al., 2018; Lee et al., 2020). Some scholars also explored whether the search for calling is associated with work meaning and job satisfaction. Empirical evidence showed that the links from searching for calling to work meaning and job satisfaction have been either significantly negative (Jaramillo, 2011; Steger et al., 2012) or positive (Doenges, 2011; Willemse and Deacon, 2015; Wang and Yu, 2018).

## The Coexistence of Searching for and Presence of Calling within Individuals

Prior studies found a close association between searching for and presence of calling, but this correlation varied across studies. Some studies indicated a positive correlation between the search for and presence of a calling (e.g., Dik et al., 2012; Domene, 2012; Willemse and Deacon, 2015; Autin et al., 2017). People who perceive a strong calling may have an active search for calling and vice versa. In this case, the search for and presence of a calling can co-exist at high levels in individuals. On the contrary, several studies indicated a negative association between them (e.g., Duffy and Sedlacek, 2007; Steger and Dik, 2009; Praskova et al., 2015; Xu and Tracey, 2017). A person may be in the state of actively searching for but not perceiving a calling, or having a strong sense of calling but low scores on seeking a calling. These inconsistent findings indicate that the combinations of the extent to which they perceive and seek a calling may vary from person to person.

Moreover, some scholars suggested that the notion of self-identity and self-development are central to the concept of calling (Elangovan et al., 2010; Hirschi, 2011). It implies that a calling's development process might be understood in conjunction with identity development. According to the identity status model proposed by Marcia (1966), identity formation can be understood along with two dimensions of exploration and commitment. Exploration refers to the degree of active questioning and considering various identity alternatives for one's goals, values, and convictions, while commitment is defined as the degree to which people have made invested choices about important identity-related issues (Luyckx et al., 2009, 2010). These two dimensions are concurrent within individuals and can be joined to produce different patterns of identity formation (Luyckx et al., 2009, 2010). In the search process of calling, people need to continually explore themselves and the external environment to discern or develop their calling (e.g., Elangovan et al., 2010; Xu and Tracey, 2017). The presence of calling is closely related to individuals' self-clarity and the commitment to their self-chosen career (e.g., Duffy and Sedlacek, 2007; Duffy et al., 2018). That is, the search for calling might function as identity exploration, while the presence of calling might function as identity commitment. They are not isolated within individuals but concurrent in a combined manner.

As mentioned above, searching for and presence of calling can co-exist within individuals rather than being isolated. However, prior relevant studies regarded searching for calling as an isolated stage from the presence of calling and neglected individuals' presence of calling when exploring the effect of searching for calling, which might lead to the conflicting findings in the search for calling. Given the close association between calling and meaning in life (Dik and Duffy, 2009), some researchers found that SOM and POM could co-occur and interact, such as the mitigation of POM on the negative effects of SOM (Steger et al., 2009a; Park et al., 2010; Yek et al., 2017). It indicates that the presence of calling may influence the link of searching for calling to its psychosocial functioning outcomes. That is, how searching for calling relates to its psychosocial functioning outcomes might be different depending on the strength of perceiving a calling.

## The Person-Centered Approach in the Calling-Related Domain

The variable-centered methods can apply moderated regression analysis to examine how the search for and presence of calling interact to influence people's psychosocial functioning. However, the configurations of searching for and presence of calling obtained from the moderating regression analysis may not exist in reality (Morin et al., 2011). In turn, the combination types between the search for and presence of calling existing in the real situation may also show a non-significant moderation result that the association between searching for calling to its outcomes would be not influenced by the presence of calling. Moreover, the variable-centered methods assume that the whole population can be described by a single set of “averaged” parameters, which most likely oversimplify reality (Hofmans et al., 2020).

Aware of the above limitations of variable-centered methods, more and more researchers advocate person-oriented methods to supplement these limitations and rethink research issues (e.g., Debowska et al., 2017, 2018; Morin et al., 2018). Latent profile analysis (LPA) is an emerging person-oriented method to interpret how some continuous variables combine conjointly within individuals. LPA assumes population heterogeneity and aims to identify latent subgroups within a population by a set of continuous variables (Debowska et al., 2017; Boduszek et al., 2019; Hofmans et al., 2020). Compared with the traditional person-centered method (e.g., cluster analysis), LPA can provide a more accurate and objective classification based on a formal model (Magidson and Vermunt, 2002; Hofmans et al., 2020). LPA has been widely used to explore distinct configurations of multiple characteristics that are close to the actual situation, and further reveal how these characteristics work together to influence individuals' psychosocial functioning (Hirschi and Valero, 2015; Meyer et al., 2015; Perera and McIlveen, 2017; Mäkikangas, 2018).

Despite no direct empirical evidence on the profiles of searching for and presence of calling and their effects on people's psychosocial functioning, prior studies about the SOM and POM provide some implications. Some researchers utilized a person-oriented approach to investigate the meaning-in-life profiles and their effects on people's psychosocial functioning (e.g., life satisfaction, psychological, and eudaimonic well-being). For instance, Dezutter et al. (2014) found that in a sample of American emerging adults, five distinct profiles emerged: high POM high SOM, undifferentiated (average POM and average SOM), high POM low SOM, low POM high SOM, and low POM low SOM; Moreover, emerging adults with profiles high on the level of POM (i.e., high POM high SOM and high POM low SOM) showed the most adaptive psychosocial functioning, whereas those with profiles low on the level of POM (e.g., low POM high SOM and low POM low SOM) reported maladaptive psychosocial functioning. The comparison between some profiles showed a moderating effect of POM on the effect of SOM, partly similar to prior variable-oriented studies. For instance, empirical evidence showed a significant interaction of SOM and POM to well-being and health outcomes. Individuals with both high SOM and POM would feel lower health anxiety than those with high SOM and low POM (Yek et al., 2017), and even experience more well-being than those with high POM and low SOM (Steger et al., 2009a; Park et al., 2010).

## The Present Study

Overall, the present study aims to conduct LPA among the Chinese teacher sample to explore the possible combinations of searching for and presence of calling as well as these latent profiles' differences in the psychosocial functioning outcomes. The present study chooses work meaning and job satisfaction to reflect people's psychosocial functioning. As prior studies showed, work meaning and job satisfaction have received a relatively high amount of attention but show conflicting relationships with searching for calling across different studies (e.g., Jaramillo, 2011; Steger et al., 2012; Willemse and Deacon, 2015; Wang and Yu, 2018). So, this study aimed to explore how different configurations between the search for and presence of calling are associated with work meaning and job satisfaction, revealing whether the effect of searching for calling on work meaning and job satisfaction is related to the presence of calling. Because prior studies were mostly conducted in Western culture and the lack of diversity would impede us from knowing more about calling in Non-Western cultures (Duffy and Dik, 2013), it is necessary to examine calling-related issues in the Chinese context. Additionally, the teacher is an occupation that is easier to find and develop calling than some other occupations (e.g., salespersons, factory workers; Wrzesniewski et al., 1997; Peng et al., 2019). It is suitable to explore the calling profiles with their related outcomes within the teacher sample. Moreover, society requires teachers with strong callings because their job tasks are closely related to children's growth and development. Examining teachers' calling profiles can help us know better about teachers' development of calling and how to enhance their work meaning and job satisfaction, which has practical significance.

As mentioned above, prior empirical studies have found different combinations between SOM and POM as well as their different effects on people's adaptive psychosocial functioning, showing that POM can mitigate the negative effects of SOM or enlarge the positive effects of SOM. Specifically, among those who already had substantial meaning in their lives, the more actively they search for meaning, the greater life satisfaction, more happiness, and less depression they experience (Steger et al., 2009a; Park et al., 2010; Yek et al., 2017). Analogously, among those who already have a strong sense of calling toward their career, the search for calling would be positively associated with people's adaptive psychosocial functioning (i.e., work meaning and job satisfaction). As such, the present study hypothesizes that the search for and presence of calling may combine to at least two different groups. For instance, both the search for and presence of calling are high levels in one profile, and the presence of calling is high but the search for calling is low in one profile. Furthermore, the present study hypothesizes that work meaning and job satisfaction may differ across the distinct calling profiles. For instance, individuals who endorsed both high levels of searching for and presence of calling may experience more meaningfulness at work and satisfaction to their job than those with high presence of and low search for calling.

## Method

### Participants and Procedure

A total of 809 kindergarten teachers were invited to complete this questionnaire. The data of 83 participants were excluded because (a) they respond to items faster than the rate of two seconds per item (Huang et al., 2012), or (b) their response to the validity test item was questionable (i.e., “Please choose the second choice ‘somewhat disagree’ in this item”), or (c) they filled the questionnaire with obvious response patterns (i.e., selecting items on the same answer or a certain regularity). Overall, 726 participants (89.74%) were included in further analysis. Among the valid sample, most participants were female (97.8%), in line with the country gender ratio of kindergarten teachers surveyed by the Chinese Ministry of Education. The participants' age ranged from 20 to 60 years old, and the average age was 32.51 (*SD* = 7.61). Participants reported a mean work experience of 11.04 years (*SD* = 8.38), ranging from 1 to 44 years. Regarding the education levels, 26 (3.6%) had completed high school or less, 676 (93.1%) had an undergraduate degree, and 24 (3.3%) had a master's degree or above.

This study's ethical approval was granted by the Ethics Committee of Psychology school at all authors' institutions. Participating kindergartens were purposively selected based on geographic location, divided into seven regions (e.g., East China, South China, Central China). Meanwhile, all participating kindergartens' headmasters are engaging in a country training project conducted by this study's corresponding author, facilitating this investigation. With these kindergarten headmasters' help, a questionnaire link was delivered to kindergarten teachers via their headmasters. Before the survey, all kindergarten teachers were provided with the written informed consent and instructions on how to complete the questionnaire. They were also informed that participation was voluntary and ensured that their responses would be anonymous and confidential.

### Measures

#### Calling

We applied the Chinese adaptation (Zhang et al., 2015a) of the Brief Calling Scale (BCS; Dik et al., 2012) to measure kindergarten teachers' search for and presence of a calling toward their career. It consists of two sub-scales named the search for and presence of calling, each containing two items. Search for calling describes the extent to which individuals seek a calling (e.g., “I am searching for my calling as it applies to my career”). Presence of calling describes the extent to which individuals have a calling (e.g., “I have a calling to a particular kind of work”). All items were rated on a 5-point Likert scale from 1 (not at all true of me) to 5 (totally true of me). Higher scores indicate the stronger search for and presence of calling. The validity of the search for and presence of calling has been supported in the findings of their close association with criterion variables, such as intrinsic work motivation and meaning in life (Dik et al., 2012). Moreover, previous studies showed the expected correlation among the Chinese adapted scale, other calling measures, and some criteria variables (e.g., career/life satisfaction; Zhang et al., 2015a,b). The current study found that the two items of BCS-search correlate at *r* = 0.78, and the two items of BCS-presence correlate at *r* = 0.78.

#### Work Meaning

Work meaning was assessed with the five-item (e.g., “The work that I do is important”) scale developed by Bunderson and Thompson (2009). Participants rated the items on a 7-point Likert scale ranging from 1 (strongly disagree) to 7 (strongly agree). Higher scores indicate perceiving a stronger sense of meaning at work. Bunderson and Thompson (2009) showed that this scale possessed acceptable psychometric properties and correlated as predicted with occupational identification, calling, and occupational importance. Additionally, prior studies found that this measure had good reliability and positive associations with prosocial motivation and task importance in Chinese workers samples (Chen et al., 2016). In the current study, the scale reached a Cronbach's alpha of 0.91.

#### Job Satisfaction

The job satisfaction subscale of the Michigan Organizational Assessment Questionnaire was applied to measure individuals' overall job satisfaction (Cammann et al., 1983). It consists of three items (e.g., “All in all, I am satisfied with my job”), rated on a 7-point Likert scale ranging from 1(strongly disagree) to 7 (strongly agree). The higher scores one obtains, the stronger their satisfaction with their job. Studies showed that this scale has high reliability and positive associations with calling, work engagement, and life satisfaction as expected (Gazica and Spector, 2015). Moreover, in Chinese samples, this scale has been demonstrated to have acceptable reliability and validity (e.g., Tu et al., 2017; Alkhadher et al., 2020). In the current study, the Cronbach's alpha of this scale was 0.91.

### Statistical Analysis

The statistical analysis comprised three phases. Firstly, some preliminary analyses were conducted. The analysis of missing values and multivariate normality test were implemented by SPSS 24.0. Meanwhile, SPSS 24.0 was used to examine the appropriateness of factor analysis. It indicates the correlation matrix is appropriate for factor analysis when the Kaiser-Meyer-Olkin (KMO) measure of sampling adequacy is above 0.80, the measures of sampling adequacy (MSA) in all the off-diagonal elements of the anti-image correlation matrix are above 0.50, and the Bartlett's test of sphericity shows a clear rejection of the independence hypothesis (Dziuban and Shirkey, 1974; Shanthi, 2019). The confirmatory factor analyses (CFA) were conducted by AMOS 23.0 to validate all measures' psychometric properties. In the CFA, model fit was assessed by the Comparative fit index (CFI), the Tucker-Lewis Index (TLI), the Root Mean Square Error of Approximation (RMSEA), and the Standardized Root Mean Square Residual (SRMR). Values above 0.95 for CFI and TLI, below 0.08 for SRMR, and close to or below 0.06 for RMSEA indicate a good fit (Hu and Bentler, 1999).

Secondly, the latent profile analyses (LPA) was conducted to extract kindergarten teachers' profiles, using the robust maximum likelihood (MLR) estimator in Mplus 8.3. To avoid local solutions, all LPA were performed with 3,000 random sets of start values, 1,000 iterations, and 200 best solutions retained for final stage optimization (Geiser, 2013; Meyer et al., 2015; Spurk et al., 2020). When selecting the best fitting profile solution, multiple statistical fit values and content decision criteria were applied. Model fit values used in the present study included the Akaike Information Criterion (AIC), the Bayesian Information Criterion (BIC), the sample-adjusted BIC (SaBIC), the adjusted Lo-Mendell-Rubin Test (LMR), the Bootstrapped Likelihood Ratio Test (BLRT), and Entropy. Lower values for the AIC, BIC, and SaBIC and higher entropy values above the cut-off of 0.80 indicate a better fit of the model to data (Spurk et al., 2020). The LMR and BLRT evaluate whether a k+1-profile model fits better compared with a k-profile solution. Additionally, the theoretical meaningfulness and the parsimony of profiles are considered content decision criteria (Spurk et al., 2020).

Finally, after identifying the final profile solution, the BCH method in Mplus (Asparouhov and Muthén, 2014) was applied to compare the retained profiles' differences in work meaning and job satisfaction. It provides equality tests of class-specific means of the distal outcomes across groups by conducting Wald tests (Bakk and Vermunt, 2016). Researchers found that the BCH method outperformed the three-step approach and DCOM command which are also two widely used methods to explore the relationship between the latent categorical variable and other auxiliary observed variables (Asparouhov and Muthén, 2014).

## Results

### Preliminary Analysis

All participants had no missing data on all main study variables. No variables had absolute skewness or kurtosis values above 3, and all appeared normally distributed on the histogram and P-P diagram. Results about the appropriateness of factor analysis showed that the matrix is generally appropriate for factor analysis [KMO = 0.894; Bartlett's test of sphericity: χ^2^ (66) = 6677.129, *p* < 0.001; Items' MSAs were ranging from 0.60 to 0.96]. In terms of CFA results, the test of the four-factor model, including the search for, presence of calling and two criteria-related outcomes (i.e., work meaning and job satisfaction), showed an excellent fit to the data (χ^2^ = 152.739, *df* = 48, CFI = 0.972, TLI = 0.961, RMSEA = 0.055, and SRMR = 0.025), and all indicators loaded onto their respective factors above values of 0.74, supporting the four included constructs as separate variables in our analysis. Table 1 displays the descriptive statistics and correlations for all the observed variables.

**Table 1:** Descriptive statistics and correlations for all the observed variables.

**Variable**	***M***	***SD***	**1**	**2**	**3**	**4**
1. BCS-S	3.70	1.03	-			
2. BCS-P	4.15	0.80	0.26	-		
3. Work meaning	6.17	0.84	0.19	0.61	-	
4. Job satisfaction	5.94	1.10	0.13	0.57	0.79	-

*Note. N = 726. All the above correlations are significant at p < 0.001*.

### Profiles of Calling

Table 2 showed the fit indices for solutions with two to six. The BLRT was not useful in selecting the optimal solution. Although the AIC, BIC, and SaBIC values kept descending with additional profiles, the non-significant adjusted LRT when continuing to six profiles indicated that the six-profile solution was not superior to the five-profile solution and the latter one should be retained. However, in terms of content decision criteria, the additional profile from four profiles to five profiles, characterized by the low search for calling and high presence of calling (*M*_BCS−S_ = 2.11, *M*_BCS−P_ = 4.10), had only minor quantitative level differences with profile 2 in the four-profile solution. And this additional group has a class size less than 50 (*n* = 33), making the trustworthy generalization doubtful (Muthen and Muthen, 2000). Due to reasons of parsimony, the four profiles should be retained rather than the five-profile model. Moreover, the four-profile solution had great classification accuracy with a high entropy value of 0.96, above the recommended threshold levels of 0.80 (Spurk et al., 2020). In the four-profile solution, the average posterior probabilities of class membership in the domain profile varied from 0.948 to 0.998 (*M* = 0.977), with generally low cross-probabilities ranging from 0.000 to 0.051 (*M* = 0.019). To sum up, the four-profile model was retained as the final latent profile solution of calling.

**Table 2:** Fit results and class sizes for the latent profile analysis.

**Model**	**LL**	**FP**	**AIC**	**BIC**	**SaBIC**	**Entropy**	**LMR (*p*)**	**BLRT(*p*)**	**Class sizes**
2-Profile	−1872.84	7	3759.68	3791.79	3769.57	0.67	<0.001	<0.001	189, 537
3-Profile	−1747.02	10	3514.04	3559.92	3528.16	0.94	0.029	<0.001	314, 128, 284
4-Profile	−1635.78	13	3297.55	3357.19	3315.91	0.96	<0.001	<0.001	181, 83, 168, 294
5-Profile	−1532.10	16	3096.20	3169.60	3118.79	0.96	<0.001	<0.001	33,70, 285, 148,190
6-Profile	−1267.56	19	2573.12	2660.28	2599.95	1.00	0.508	<0.001	117, 223, 11, 261, 53, 61

*Note. N = 726. LL, Model Log-Likelihood; FP, Free Parameters; AIC, Akaike Information Criterion; BIC, Bayesian Information Criterion; SaBIC, the Sample-adjusted Bayesian Information Criterion; LMR(p), p-Value for the adjusted Lo-Mendell-Rubin test; BLRT(p), p-Value for the Bootstrapped Likelihood Ratio test*.

**Figure 1:**
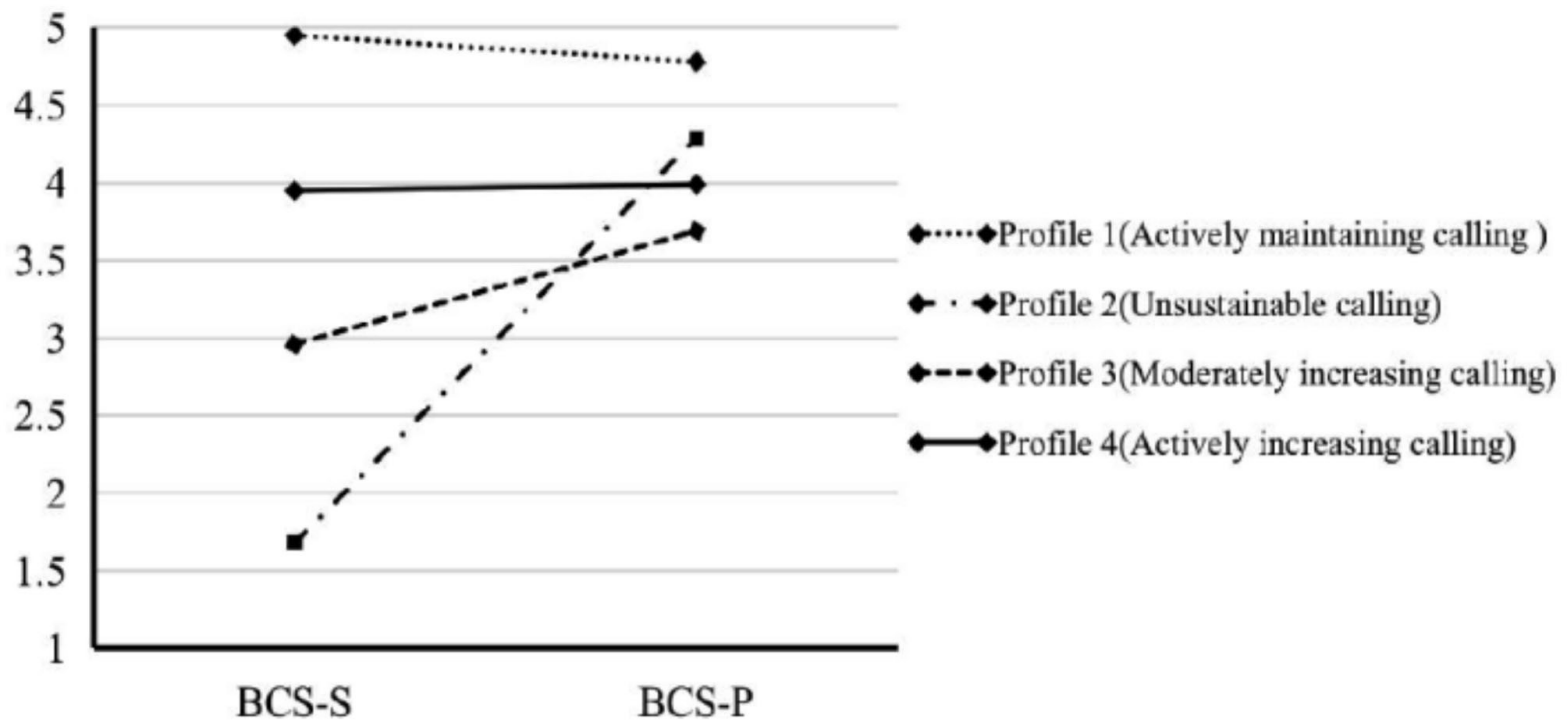
Mean scores in the indicators search for and presence of calling for the four-profile solution.

Table 3 showed the mean levels of two calling variables in the retained model, and Figure 1 depicted the means of two calling indicators for the four-profile solution. Some scholars argued that after people discover their calling, they will also continually and dynamically try to maintain or increase their experience of calling rather than ceasing the calling search (Dik et al., 2012). The first profile (*n* = 181, 24.93%) showed the highest values in the search for and presence of calling. Due that the teachers in profile 1 possessed an almost maximum level of perceiving a calling that the scores of BCS-P measure can achieve, the high levels of searching for calling are more likely to maintain the presence of calling they already had. So, we called profile 1 the *actively maintaining calling* group. The second profile, constituting 11.43% (*n* = 83) of the sample, showed a high presence of calling and the lowest values in searching for calling. Even though the teachers in profile 2 endorsed that they had a calling, the low level of searching for calling means that they are more likely to cease their calling maintaining or enhancement, which may be harmful to the sustainable development of calling. So, we called it the *unsustainable calling* group. In the third profile (*n* = 168, 23.14%), the teachers reported the moderate-level presence of and search for calling, indicating that they intended to increase the existing presence of calling but only with a moderate motivation to search for further calling. So, we named this the *moderately increasing calling* group. In the final profile with the largest size (*n* = 294, 40.50%), the teachers possessed a slightly high calling which still had further room to rise. Meanwhile, they had a high level of searching for calling, indicating that those teachers would be more likely to increase the presence of calling they already had. So, we named this the *actively increasing calling* group.

**Table 3:** Mean levels of the search for and presence of a calling in the four-profile model.

**Calling**	***M***	**Profile 1**	**Profile 2**	**Profile 3**	**Profile 4**
BCS-S	3.70 (1.03)	4.95 (0.01)	1.68 (0.05)	2.96 (0.02)	3.95 (0.01)
BCS-P	4.15 (0.80)	4.78 (0.03)	4.29 (0.11)	3.69 (0.06)	3.99 (0.04)
*N* (%)		181 (24.93%)	83 (11.43%)	168 (23.14%)	294 (40.50%)

*Note. N = 726. Profile 1: Actively maintaining calling; Profile 2: Unsustainable calling; Profile 3: Moderately increasing calling; Profile 4: Actively increasing calling*.

### Relations With Work Meaning and Job Satisfaction

**Figure 2:**
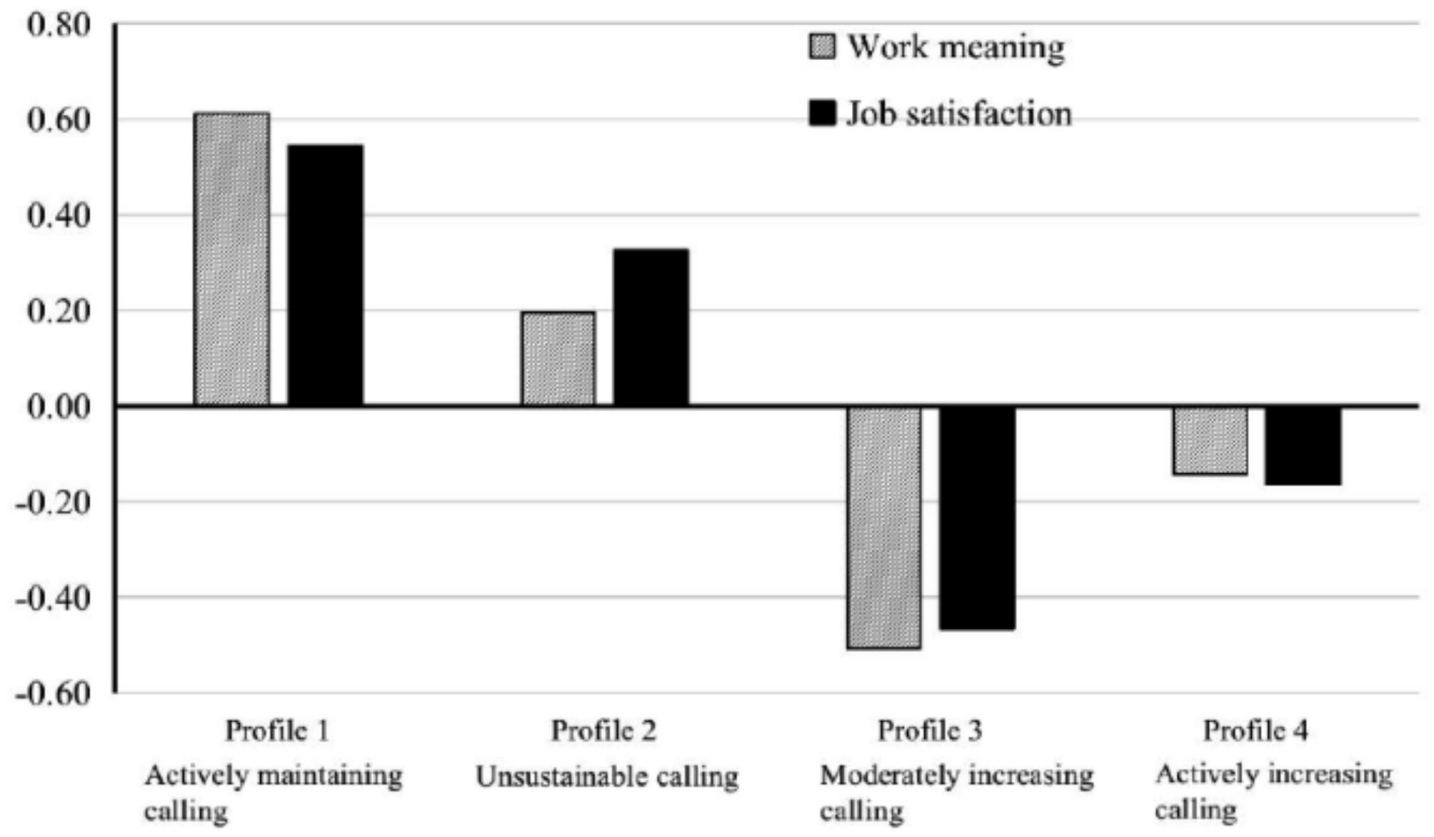
Standardized means of the correlates by the four calling profiles.

Table 4 displayed the results of the four profiles' comparisons on work meaning and job satisfaction. Figure 2 presented the standardized means of work meaning and job satisfaction for the four profiles. The results of Wald tests showed that work meaning and job satisfaction significantly differed across the four profiles (*p* < 0.001). Specifically, teachers in profile 1 (*actively maintaining calling*) experienced the highest levels of work meaning, followed by those in profile 2 (*unsustainable calling*), profile 4 (*actively increasing calling*), and profile 3 (*moderately increasing calling*) in a significantly decreasing sequence. Additionally, teachers in profiles 1 (*actively maintaining calling*) and 2 (*unsustainable calling*) had similar strengths in job satisfaction, both of whom were more satisfied with their job than those in profile 4 (*actively increasing calling*) and then followed by profile 3 (*moderately increasing calling*).

**Table 4:** Equality tests of work meaning and job satisfaction across latent profiles.

	**Profile 1(a)**	**Profile 2(b)**	**Profile 3(c)**	**Profile 4(d)**	**χ^**2**^(*df* = 3)**
Work meaning	6.69_bcd_	6.33_acd_	5.74_abd_	6.05_abc_	206.88^***^
Job satisfaction	6.55_cd_	6.30_cd_	5.43_abd_	5.76_abc_	152.12^***^

*Note. N = 726. Subscripts denote profiles which differ significantly at p < 0.05. Chi-square indicates the significance of the overall difference test. Profile 1: Actively maintaining calling; Profile 2: Unsustainable calling; Profile 3: Moderately increasing calling; Profile 4: Actively increasing calling*.

*** *p < 0.001*.

## Discussion

The current study is the first research to examine how the search for and presence of calling combine to affect people's work meaning and job satisfaction by applying latent profile analysis. *Via* this person-centered method, four calling profiles with meaningful differences emerged in the present kindergarten teachers sample. Furthermore, the results found that these four profiles differed in levels of work meaning and job satisfaction. As this study's findings indicated, kindergarten teachers in profile 2 (unsustainable calling) with the lowest search for but a high presence of calling experienced higher work meaning and job satisfaction than those in profiles 3 and 4. It shows the trends that people with a stronger presence of calling but lower searching for calling would experience more work meaning and job satisfaction. Compared kindergarten teachers with both the highest searching for and presence of calling (profile 1) to other groups of kindergarten teachers, and compared kindergarten teachers who had the slightly high search for and presence of calling (profile 4) to those with the moderate search for and presence of calling (profile 3), both of the former experienced more meaningfulness at work and/or were more satisfied with their job than the latter. In that case, it shows the trends that people with stronger searching for calling would experience more work meaning and job satisfaction. The two opposite effects of searching for calling on work meaning and job satisfaction emerging in a sample are related to participants' strength of perceiving a calling. When considering the presence of calling simultaneously, it showed that among kindergarten teachers who already perceive intensive calling, actively searching for calling is positively associated with more work meaning and job satisfaction. Similar to the interaction of SOM and POM showed in prior studies (Steger et al., 2009a; Park et al., 2010), individuals with both high searching for and presence of calling would not experience lower work meaning and job satisfaction than those with high presence of calling and low searching for calling. In sum, these findings reaffirm the positive effect of perceiving a calling on work meaning and job satisfaction. Meanwhile, they indicate that the strength of perceiving a calling can influence the effect of searching for calling, which is in line with our expectations.

Why was the active search for calling associated with more work meaning and greater job satisfaction among those who already had intensive calling toward a specific work? Studies about the interaction of searching for and presence of meaning suggested that people's existing meaning may provide a foundation for the further meaning search, which would be a process of meaning modification and expansion (Park et al., 2010). Whereas the search for meaning without the presence of meaning requires a lot of resource investment, which may be stressful and difficult (Park et al., 2010). Similarly, some researchers suggested that the development of calling was an ongoing process rather than a one-time event and needed people to continue seeking ways to maintain or enhance their existing presence of calling (Dik and Duffy, 2009; Dik et al., 2012). It implied that among those who possess intensive experience of calling, the active search for calling might be a further search for their existing calling to maintain or enhance the presence of calling they already have. The coexistence of high presence of and search for calling may create a positive spiral, yielding more positive outcomes (e.g., work meaning and job satisfaction). Thus, when kindergarten teachers have a high calling and search for further calling more actively, they will obtain more work meaning and job satisfaction. It still requires more fine-grained future research into the processes involved in searching for calling to test this explanation.

Additionally, the present study found four profiles on the configurations of the presence of and search for calling. The pattern of profiles bears some resemblance to the meaning-in-life profiles found in prior studies. Specifically, when standardizing the score means of two indicators, the actively maintaining calling, the unsustainable calling, the moderately increasing calling, and the actively increasing calling profiles showed similarities with the high presence-high search, the high presence-low search, the low presence-low search, and the average presence-average search meaning profiles, respectively (Dezutter et al., 2014). To some extent, it reflects the importance of meaning in life to the development of calling from a new perspective. Moreover, the results imply that people may be at different development status of calling. Some people are continually searching for calling even though they had a clear calling, whereas some people would cease their further search for calling when they perceived a calling. For the former, three profiles that we found represent level profiles because they showed the tendency for a person to be high, medium, or low level across all indicators. For the latter, the unsustainable calling profile showed a qualitative (shape) difference in the two calling indicators (Morin and Marsh, 2015). The unsustainable calling profile was small in size, accounting for only 11.43% of participants. Thus, this profile would likely not have been detected using a variable-centered approach (Wang and Hanges, 2011). It exemplifies the advantage of using a person-centered approach in our analysis.

Among those who have an intensive sense of calling, why does someone continually search for further calling, and why does someone stop further searching? It might be closely related to people's different causes of seeking the calling. Searching for a calling has been considered as a process of searching for life meaning (Elangovan et al., 2010; Dik et al., 2012). Regarding the cause of search for life meaning, there exist deficit correcting hypothesis and life affirming hypothesis. The deficit correcting hypothesis maintains that meaning search primarily originates from its absence or deficit, and once the meaning is restored, the search would decay; On the contrary, life affirming hypothesis indicates that meaning search stems from the motivation to consolidate and strengthen life experience, and therefore the effort to pursue life meaning is always incessant (Steger, 2013; Li et al., 2018). These two hypotheses have been supported by substantial empirical evidence, and they are not entirely antagonistic (e.g., Steger et al., 2008, 2009b; Steger, 2013; Li et al., 2018). In the present study, the unsustainable calling profile reflects a cause of calling search similar to the deficit correcting hypothesis, suggesting that people would cease seeking calling once they obtain intensive calling experiences. As opposed to this, the actively maintaining calling profile shows a cause of calling search similar to the life affirming hypothesis, suggesting that people would never be satisfied with their calling search and this search could enhance or maintain their calling experiences. This conjecture needs further research to explore.

Moreover, the present study showed that the active search for calling with an intensive experience of calling would be more beneficial for one's experiences of work meaning and job satisfaction. However, only a quarter of kindergarten teachers were at the stage where the presence of and search for calling are high. It urges researchers to explore how to conjointly enhance people's degree on the presence of and search for calling, increasing the amount at the stage of actively maintaining calling. Besides, as shown in the current study, people in the unsustainable calling profile experienced more work meaning and job satisfaction than those in the moderately and actively increasing calling profiles. Prior studies have shown that the experience of calling will change over time and is difficult to sustain (Dobrow, 2013; Zhang et al., 2018). From the long-term perspective, among those who have a strong sense of calling but cease the further search, how do they maintain their already perceived calling? Without the protection of the presence of calling, does the search for calling still play a positive effect on work meaning and job satisfaction? These questions require more fine-grained future research to address and broaden our knowledge about the calling development process.

Furthermore, prior studies suggested a possibility that someone was actively searching for meaning but had not yet found meaning (Dezutter et al., 2014; Krok, 2018). However, a profile where people reported not having a calling but a high search for calling did not appear in our study sample. It might be related to the present study's sample characteristics, such as the career stage and occupation. Compared with the emerging adults explored by prior studies (Dezutter et al., 2014; Krok, 2018), the present study focuses on the working adults whose career goals are relatively clear. Besides, in Chinese culture, society always advocates teachers to view their work as calling due to job tasks' particularity. The teacher's job tasks are closely related to children's growth and development. It reflects a strong other-oriented nature, making it easier for teachers to experience their social value and life meaning. As such, teachers can find and develop calling easier than some other occupations (e.g., salespersons, factory workers; Wrzesniewski et al., 1997; Peng et al., 2019). So the samples in the present study were less likely to report no calling experience. It implies future research to explore how it might be in other career stages and occupations.

### Theoretical and Practical Implications

There are some theoretical and practical implications. In terms of theoretical implications, this study's findings enrich the understanding of the search for calling, extending previous findings on the effect of the search for calling. Prior studies considered the calling search as a resource depletion process, which yields negative outcomes (e.g., depression, indecisiveness; Duffy and Sedlacek, 2007; Steger and Dik, 2009; Dobrow et al., 2019). However, the present study suggests that search for calling does not necessarily have to be viewed in negative terms; Instead, the search for calling can play a positive role for those adults who already have calling. Moreover, the present study used the person-centered method to provide an integrative representation of searching for and presence of calling, producing unique information about the potential styles of calling development as well as the complex association between searching for and presence of calling. Regarding the practical implications, this study's findings can help managers identify the target or preferred calling profiles in terms of individuals' adaptive psychosocial functioning, such as a profile associated with the most work meaning and job satisfaction. When recruiting new teachers, the headmasters or managers consider their existing calling and activeness to search for further calling simultaneously. For example, suppose the headmasters want to enhance teachers' work meaning and job satisfaction through the selection system. In that case, it may not be the most effective approach to only focus on teachers' presence of calling because teachers who perceive strong calling but different strengths in the search for calling may differ significantly in the levels of work meaning and job satisfaction. As for the kindergarten teachers who have been working, the headmasters could set up the classification management for the teachers in different calling profiles and adopt different strategies when enhancing teachers' presence of calling and motivating their search for calling.

### Limitations and Future Research

Some limitations to the present study should be considered, which suggest avenues for future research. First, the data were collected among teachers from China, which are a female-dominated sample and a calling-salient occupation. It raises the question of whether the findings may exist in other cultures, occupations, or samples. Future research should test results replicability in other samples, such as culturally diverse samples, male-dominated or gender-neutral samples, and samples with a high challenge to discover a calling (e.g., salespersons, factory workers). Second, the present study only chose two work-related outcomes (i.e., work meaning and job satisfaction) to validate the interaction of searching for and presence of calling. Future studies can examine whether the presence of calling influences the effect of searching for calling on other outcome variables, such as work-related overt behavior (e.g., organizational citizen behavior) or well-being in general (e.g., life meaning and life satisfaction). Third, our data were cross-sectional, which will preclude causal inferences on the relations of searching for and presence of calling with its outcomes (i.e., work meaning and job satisfaction). Longitudinal designs are needed to examine the causality better. The longitudinal designs can also explore whether people would transition from one latent profile to another across time, adding more information about the development process of calling. Finally, the present study only focused on combining searching for and perceiving a calling to explore the latent profiles, excluding living a calling. Living a calling refers to “when a person has a calling and can engage in work that allows their calling to manifest on a regular basis” (Autin et al., 2017, p. 690). Previous studies examined the interaction of perceiving and living a calling on outcomes (Duffy et al., 2012; Park et al., 2016). Future research can apply a person-centered approach to explore the potential configurations of perceiving and living a calling with their outcomes or predictors, providing additional insight into the calling literature.

## Data Availability Statement

The raw data supporting the conclusions of this article will be made available by the authors, without undue reservation.

## Ethics Statement

The studies involving human participants were reviewed and approved by the Ethics Committee of Psychology school of Northeast Normal University. The patients/participants provided their written informed consent to participate in this study.

## Author Contributions

FL, RJ, and DL contributed to the idea and design of the study. RJ organized the database. FL performed the statistical analysis and wrote the manuscript. All authors contributed to manuscript revision, read, and approved the submitted version.

## Conflict of Interest

The authors declare that the research was conducted in the absence of any commercial or financial relationships that could be construed as a potential conflict of interest.
